# Transperineal Ultrasound in Chronic Pelvic Pain: A Systematic Review

**DOI:** 10.1002/jcu.70406

**Published:** 2026-09-14

**Authors:** Giulia Paoni Saccone, Noemi Tonti, Valeria Gangale, Maria Grazia Porpora, Federica Tomao, Violante Di Donato, Innocenza Palaia, Giusi Santangelo, Ludovico Muzii, Giuseppe Rizzo, Giorgia Perniola

**Affiliations:** ^1^ Department of Maternal and Child Health and Urological Sciences Policlinico Umberto I, Sapienza University of Rome Rome Italy

**Keywords:** chronic pelvic pain, pelvic floor musculature dysfunction, pelvic floor musculature morphometry, transperineal ultrasound

## Abstract

This systematic review aimed to explore the application and effectiveness of transperineal ultrasound as a diagnostic tool for evaluating pelvic floor musculature (PFM) dysfunction in patients with chronic pelvic pain and related conditions, including endometriosis, vulvodynia, and gynecological cancers. Comprehensive searches were conducted across five databases, resulting in the identification of 395 articles, of which 17 were included after screening. Studies reported significant PFM alterations, including a reduced levator hiatal area and decreased excursion of the levator plate angle and anorectal angle, indicating increased PFM tone and diminished contractile capacity. Transperineal ultrasound has shown substantial reliability in assessing PFM disorders.

## Graphical abstract

Graphical abstract illustrating the systematic review process and highlighting key pelvic floor musculature (PFM) alterations detected by transperineal ultrasound (TPU) in patients with chronic pelvic pain (CPP) and associated conditions. These findings provide insights to enhance understanding and management of CPP.

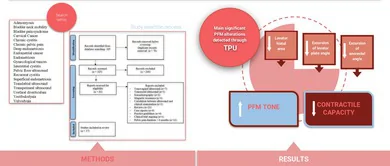

## 1 Introduction

Chronic pelvic pain (CPP) is defined as a pain with intermittent or constant characteristics that persists for at least 6 months not associated with menstruation, intercourses or pregnancy (RCOG 2012). Although it is one of the most frequent causes of outpatient access, incidence, prevalence and socio‐economic impact of CPP remain unclear (Jarrell et al. 2018). One of the most recent reviews published in literature estimates prevalence of CPP between 5.7% and 26.6% (Ahangari 2014).

CPP is a condition with a multifactorial etiology, commonly associated with visceral and musculoskeletal disorders, as well as psychosocial factors (Table 1) (Obstetrics and Gynecology 2020).

**TABLE 1:** Conditions associated with chronic pelvic pain (CPP).

Visceral	Neuromusculoskeletal	Psychosocial
Gynecologic Adenomyosis Adnexal mass Chronic pelvic inflammatory disease Endometriosis Leiomyoma Obstetric trauma Ovarian syndrome Pelvic adhesions Radiation therapy Vestibulitis Vulvodynia Gastrointestinal Celiac disease Colorectal cancer and cancer therapy Diverticular colitis Inflammatory bowel disease Irritable bowel syndrome Urologic Bladder cancer and cancer therapy Chronic or complicated urinary tract infection Interstitial cystitis Painful bladder syndrome Urethral diverticulum	Fibromyalgia myofascial syndromes Coccydynia Musculus levator ani syndrome Postural syndrome abdominal wall syndromes Muscular injury Trigger point Neurologic Abdominal epilepsy Abdominal migraine Neuralgia Neuropathic pain	Abuse Physical, emotional, sexual Depressive disorders Major depressive disorder Persistent depressive disorder (dysthymia) Substance‐induced or medication‐induced depressive disorder Anxiety disorders Generalized anxiety disorder Panic disorder Social anxiety disorder Substance‐induced ormedication‐induced anxiety disorder Somatic symptom disorders Somatic symptom disorder with pain features Somatic symptom disorder with somatic characteristics Substance use disorders Substance abuse Substance dependence

*Note:* Information sourced from Chronic Pelvic Pain: ACOG Practice Bulletin, Number 218. Obstet Gynecol. 2020 (Obstetrics and Gynecology 2020).

Recent evidence emphasizes the pivotal role of central sensitization in the maintenance of chronic pain syndromes. Alterations in brain structure and function, including enhanced activation of pain‐processing regions and dysregulation of descending modulatory pathways as well as the hypothalamic–pituitary–adrenal axis, may underlie the persistence and amplification of pain in the absence of substantial peripheral nociceptive input (Coxon et al. 2018).

As emerged in literature, contractile activity and tone of pelvic floor musculature (PFM) play a fundamental role in the pathogenesis of algic symptomatology in patients suffering from CPP syndromes impairing health and quality of life of women during reproductive age (Del Forno et al. 2020).

It is difficult to identify which muscles are related to pain syndrome, but it is well known that an altered relaxation phase or a paradoxical contractile activity may also develop other symptoms associated with CPP such as changes in defecation and urination or sexual dysfunction (Faubion et al. 2012; Mabrouk et al. 2018; Butrick 2009).

PFM anatomy and function can be evaluated through several methods. Gynecological examination with digital vaginal exploration, either unidigital or bidigital, using the modified Oxford Scale is the most common tool (Laycock 2019).

Recently, transperineal ultrasound (TPU) has been considered an accurate tool to assess pelvic floor dysfunctions (Salsi et al. 2017). TPU is a reliable, dynamic, not expensive, and reproducible diagnostic method and it can be used to perform a non‐invasive PFM assessment in patients with CPP (Jamard et al. 2020).

The objective of this systematic review is to evaluate the utility of TPU as a diagnostic tool for assessment of the pelvic floor in patients suffering from CPP.

## 2 Materials and Methods

### 2.1 Protocol, Search Strategy and Selection Process

This systematic review was performed according to the Preferred Reporting Items for Systematic Reviews and Meta‐Analysis (PRISMA) statements (Moher et al. 2009; Rethlefsen et al. 2021). A literature search of the databases PubMed, Medline, Science‐Direct, EMBASE, and Cochrane Central Register of Controlled Trial was electronically conducted and updated through July 2025. Medical subject heading (MeSH) terms, key words and word variants for “transperineal ultrasound” and “chronic pelvic pain” were searched and were used in all possible combinations using Boolean operator (Table 2). An additional search for conference abstracts was performed. Article citations were manually checked for additional references. Titles and abstracts were screened for relevance and assessed for eligibility. After the first screening phase, full texts of all articles included were evaluated (Supplementary Appendix A). Because of the recent application of TPU in routine gynecological evaluation, no publication date limit was imposed. The review protocol was not registered on any platform. Nevertheless, the review was conducted following recognized methodological guidelines to ensure transparency and rigor.

**TABLE 2:** Search strategy terms.

Adenomyosis
Bladder neck mobility
Bladder pain syndrome
Cervical Cancer
Chronic cystitis
Chronic pelvic pain
Deep endometriosis
Endometrial cancer
Endometriosis
Gynecological tumors
Interstitial cystitis
Pelvic floor ultrasound
Recurrent cystitis
Superficial endometriosis
Translabial ultrasound
Transperineal ultrasound
Urethral diverticulum
Vestibulodynia
Vulvodynia

### 2.2 Inclusion and Exclusion Criteria

Only studies using TPU to assess the pelvic floor in patients with CPP were included in this review. Randomized controlled trials, observational studies, cohort studies, conference abstracts, and case series were included. Only full‐text articles were considered for this review. Only English language articles were included.

Meta‐analyses, reviews, case reports, correspondences, personal opinions, and animal studies were excluded. All studies with assessment of CPP with other diagnostic tools were excluded. All studies in which the same population and outcomes were analyzed to avoid data overlap were excluded from this review.

### 2.3 Data Extraction and Risk of Bias

Two investigators (G.P.S. and N.T.) independently identified records from database search and removed duplicates. Disagreement was resolved between the two reviewers through discussion and in case of doubt a third author (G.P.) has been consulted. Full‐text copies of all the articles were obtained and the two investigators independently extracted relevant data. For this review the following data were extracted: first author, year of publication, type of study, number of patients, type of ultrasound probe, how the examination was conducted (at rest, under contraction and/or with Valsalva maneuver, that is a forced expiration against a closed glottis to increase intra‐abdominal pressure), symphysis‐levator distance, levator hiatal area (LHA), levator plate angle, levator plate length, ano‐rectal angle, bladder neck position and anteroposterior diameter (Table 3). Data were collected in an Excel spreadsheet. To determine the transparency of evidence synthesis results and findings and reduce risk of bias, the search strategy was based on the patient, intervention, comparison, outcome (PICO) framework (Figure 1) (Richardson et al. 1995; Eriksen and Frandsen 2018) and the ROBIS tool was used (Whiting et al. 2016).

**TABLE 3:** Ultrasound measurements description.

Ultrasound measurements	Definition	Unit	Imaging plane
Symphysis‐levator distance	Distance between the posteroinferior margin of the pubic symphysis and the anterior insertion of the levator ani muscle, located just posterior to the anorectal junction.	mm	Mid‐sagittal
Levator Hiatal Area (LHA)	Area enclosed by the levator ani muscles at the level of minimal anteroposterior diameter, including urethra, vagina, and rectum. It is measured in the plane corresponding to the plane of minimal hiatal dimensions, reconstructed from 3D/4D transperineal ultrasound volumes.	cm^2^	Axial
Levator Plate Angle (LPA)	Angle between the levator ani muscle and a horizontal reference line through the inferior margin of the symphysis. It is measured by the intersection of this horizontal reference line with a line drawn from the posteroinferior margin of the pubic symphysis to the pivotal point of the anorectal angle.	degrees (°)	Mid‐sagittal
Levator Plate Length (LPL)	Linear distance between the pubic symphysis and the posterior insertion of the levator muscle at the level of anorectal junction.	mm	Mid‐sagittal
Ano‐Rectal Angle (ARA)	Angle between the longitudinal axes of the anal canal and rectum. Identified as the angle formed at the anorectal junction.	degrees (°)	Mid‐sagittal
Bladder Neck Position	Vertical distance between the bladder neck and the inferior border of the pubic symphysis, assessed at rest and during functional maneuvers.	mm	Mid‐sagittal
Antero‐Posterior Diameter (AP)	Distance from the posteroinferior margin of the symphysis to the most anterior point of the anorectal angle, measured in the plane of minimal hiatal dimensions.	mm	Mid‐sagittal

**FIGURE 1:**
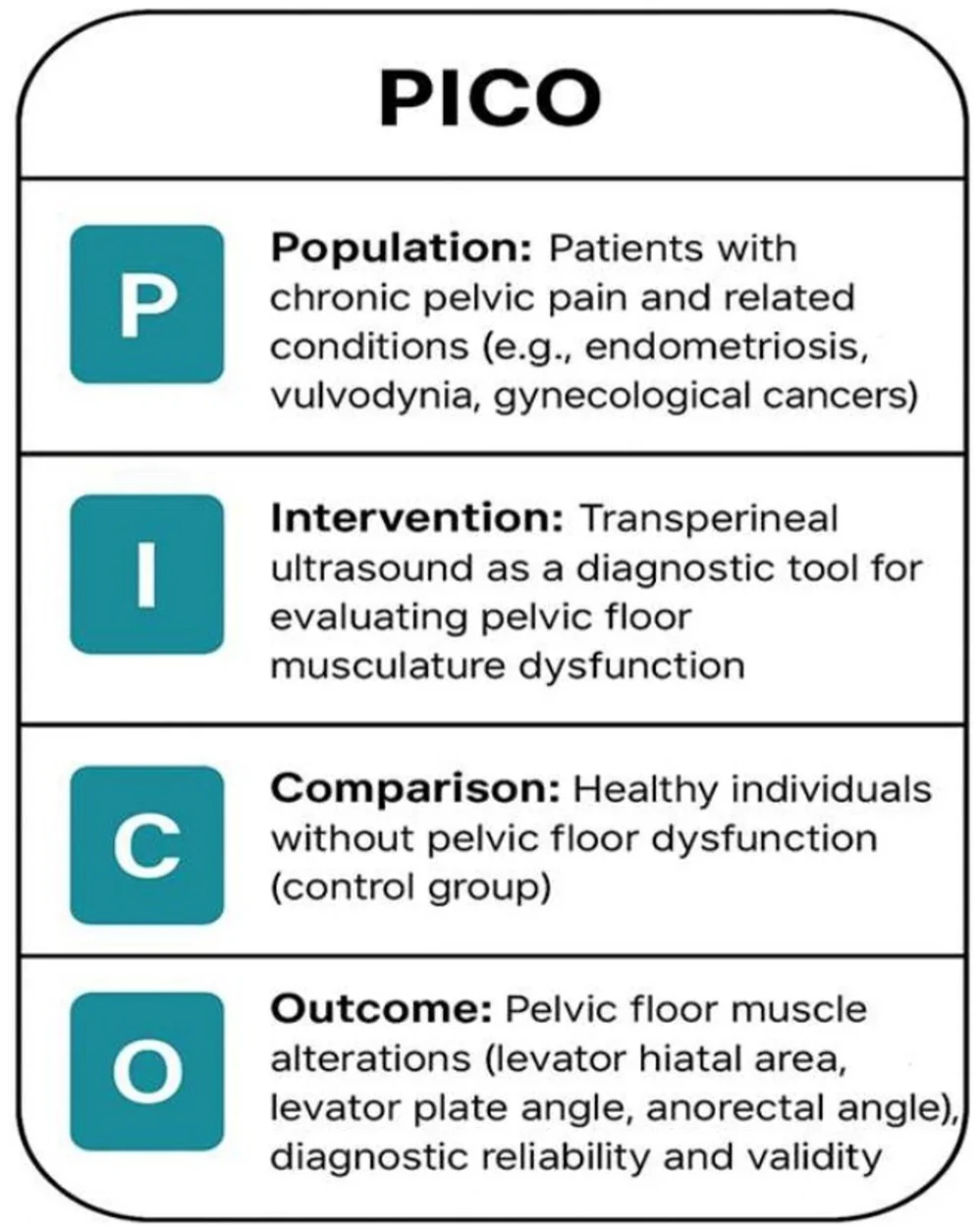
PICO framework.

## 3 Results

### 3.1 Study Characteristics

In this review 395 articles were identified. After removing duplicates, the remaining 325 studies were screened for eligibility based on the title and Abstract. 81 publications were screened in the full‐text versions, and 17 studies were included in the systematic review (Figure 2). The studies included were as follows: 6 prospective observational cohort, 5 prospective control–case, 1 prospective randomized control–case, 2 prospective randomized single‐blind control–case, 2 cross‐sectional, 1 prospective interventional. Main characteristics of the included studies are summarized in Table 4.

**FIGURE 2:**
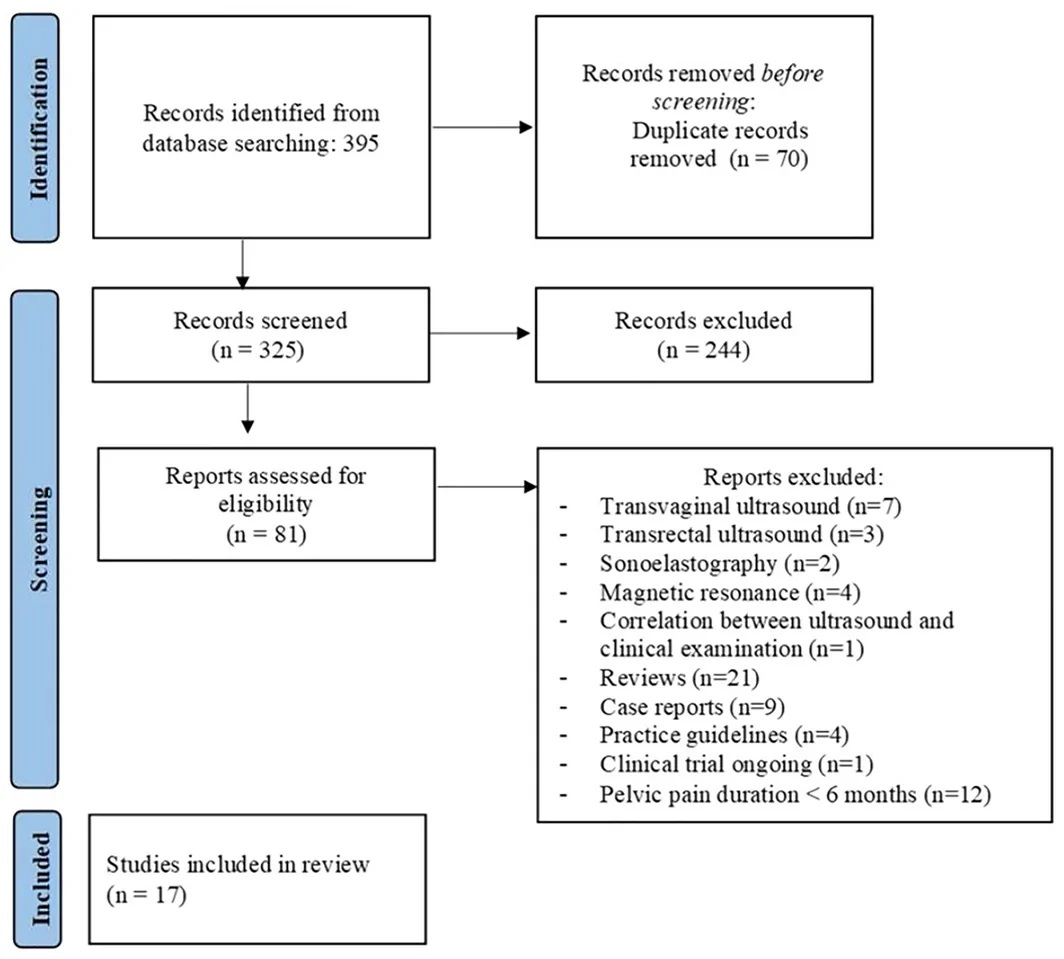
PRISMA flowchart.

**TABLE 4:** Studies characteristics.

References	Objective	Design	Sample size	Observers	Ultrasound technique	Ultrasound assessment
Raimondo et al. 2017 (Raimondo et al. 2017)	Evaluate the static and dynamic morphometry of PFM using 3D‐4D TPU in women with DIE compared with asymptomatic controls.	Observational prospective case–control	Cases: 50 nulliparous women with DIE Controls: 35 healthy nulliparous women	Single	3D/4D TPU at rest, on maximum PFM contraction and on maximum Valsalva maneuver.	LHA, LPA, LRT
Mabrouk et al. (2018)	Assess the impact of retroperitoneal infiltration on PFM function in patients with ovarian endometriosis and DIE.	Observational prospective cohort study	114 nulliparous with endometriosis (75 with DIE; 39 without DIE)	Multiple	3D/4D TPU at rest, on maximum PFM contraction and on maximum Valsalva maneuver.	LHA, LPA, LRT
Raimondo et al. (2022)	Assess 3D/4D pelvic floor US findings and chronic constipation (CC) in women with endometriosis.	Observational prospective cohort study	87 women divided into two groups: 29 (33%) with CC and 58 (67%) without CC	Single	3D/4D US at rest, on maximum PFM contraction and on maximum Valsalva maneuver.	LHA, LPA
Mabrouk et al. (2019)	Evaluate the correlation between the presence of voiding dysfunction (VD) and altered PFM morphometry in patients with posterior DIE.	Observational prospective cohort study	108 symptomatic women, divided in two groups: 48 with VD, 60 without VD	Double	3D/4D US at rest, on maximum PFM contraction and on maximum Valsalva maneuver.	LHA, LPA, LRT
Mabrouk et al. (2020)	Investigate the correlation between superficial dyspareunia and PFM morphometry.	Observational prospective cohort study	160 nulliparous sexual active divided in two groups: 81 with DIE and 79 with ovarian endometriosis (OE)	Double	3D US at rest.	LHA
Arena et al. (2022)	Assess the prevalence of the different categories of the 4‐point contraction scale in endometriotic patients vs. controls.	Observational prospective case–control	164 patients (82 DIE; 82 OE); vs. 82 healthy controls	Single	3D/4D US at rest, on maximum PFM contraction.	LHA, LPA
Del Forno et al. (2020)	Evaluate the effects of pelvic floor physiotherapy (PFP) among women with DIE and associated dyspareunia.	Observational prospective cohort study	10 nulliparous with DIE and dyspareunia	Double	3D/4D US at rest, on maximum PFM contraction and on maximum Valsalva maneuver.	LHA
Del Forno et al. (2021)	Evaluate the effect of PFP on LHA during Valsalva maneuver, using TPUS in women with DIE and superficial dyspareunia.	Randomized control trial	30 patients with DIE and superficial dyspareunia (17 study group vs. 13 control)	Double	3D/4D US at rest, on maximum PFM contraction and on maximum Valsalva maneuver.	LHA
Morin et al. (2014)	Compare PFM morphometry in women suffering from provoked vestibulodynia (PVD) to asymptomatic healthy controls.	Observational prospective case–control	51 asymptomatics vs. 49 with PVD	Single	3D/4D US at rest, on maximum PFM contraction.	LHA, LPA, LRT, ARA, displacement of the bladder neck.
McLean et al. (2016)	Determine differences in PFM between women with and without PVD.	Observational prospective case–control	38 patients with PVD vs. 39 controls	Single	3D/4D US at rest, on maximum PFM contraction and on maximum Valsalva maneuver.	LPL, LPA, ARA and displacement of the bladder neck
Thibault‐Gagnon et al. (2016)	Determine the nature of PFM involvement in women with PVD.	Observational prospective case–control	38 patients with PVD vs. 39 controls	Single	3D/4D US at rest, on maximum PFM contraction and on maximum Valsalva maneuver.	LHA, LPA, LRT
Fontaine et al. (2018)	Assess and compare PFM and function in women with primary and secondary PVD.	Observational prospective cohort study	212 patients with PVD (75 primary, 137 secondary)	Single	3D/4D US at rest, on maximum PFM contraction.	LHA, LPA, LRT, ARA and displacement of the bladder neck
Bardin et al. (2019)	Evaluate biometric changes of PFM of women diagnosed with vulvodynia submitted to physical therapy.	Single‐blinded randomized controlled trial	86 women with vulvodynia randomized either into oral medication with tricyclic antidepressant (TCA) vs. both TCA and kinesiotherapy	Single	4D‐TLUS at rest, on maximum voluntary PFM contraction and on valsalva maneuver.	LHA, LPA, ARA, LRT, AP and area of puborectalis muscle
Bardin et al. (2020)	Evaluate biometric changes in PFM in women with vulvodynia undergoing kinesiotherapy treatment protocol (KTP).	Single‐blinded randomized controlled trial	57 women with vulvodynia: 30 submitted to KTP plus TCA; 27 submitted to TCA only	Single	4D‐TLUS at rest and on maximum voluntary PFM contraction.	LHA, LPA, ARA, symphysis‐levator distance
De Morais Siqueira et al. (2021)	Assess anatomical changes in the PFM of patients with cervical or endometrial cancer underwent pelvic radiotherapy, using 4D‐TLUS.	Cross‐sectional study	64 women (26 with cervical cancer and 38 with endometrial cancer).	Multiple	4D‐TLUS at rest and on maximum voluntary PFM contraction.	LHA, LPA, puborectalis muscle thickness, puborectalis strain and LAM integrity
Cyr et al. (2021)	Characterize and compare PFM function and morphometry between gynecological cancer survivors with dyspareunia and asymptomatic women.	Comparative Cross‐Sectional Study	24 gynecological cancer survivors with dyspareunia and 32 women with a history of total hysterectomybut without pelvic pain	Single	3D/4D TPUS at rest and on maximum voluntary PFM contraction.	LHA, LPA, ARA and bladder neck position
Cyr et al. (2022)	Investigate the changes in PFM morphometry and function after multimodal physiotherapy in gynecological cancer survivors with dyspareunia.	Prospective interventional study	31 gynecological cancer survivors with dyspareunia	Single	3D/4D TPUS at rest and on maximum voluntary PFM contraction.	LHA, LPA, ARA and bladder neck position

Abbreviations: AP, antero‐posterior diameter; ARA, anorectal angle; DIE, deep infiltrating endometriosis; LHA, levator hiatal area; LPA, levator plate angle; LPL, levator plate length; LRT, left–right transverse diameter; PFM, pelvic floor musculature; TPU, transperineal ultrasound.

### 3.2 Risk of Bias Assessment

The methodological quality of the included studies was assessed using the ROBIS tool (Whiting et al. 2016). Most studies were judged to have a low risk of bias across all four domains: eligibility criteria, selection process, data collection and appraisal, and synthesis of findings. Two studies (Del Forno et al. 2020; Cyr et al. 2021) were rated as having a moderate risk of bias in the selection process domain, primarily due to small sample sizes and limited clarity in participant selection. No studies were judged to have a high overall risk of bias.

### 3.3 Endometriosis

The utility of TPU in patients suffering from endometriosis and CPP has been investigated in 8 selected studies (Raimondo et al. 2017, 2022; Mabrouk et al. 2018, 2019, 2020; Arena et al. 2022; Del Forno et al. 2020, 2021). Results are summarized in Table 5.

**TABLE 5:** Pelvic floor muscle (PFM) morphometry on three‐ and four‐dimensional transperineal ultrasound (3D–4D TPU) at rest, on PFM contraction and on Valsalva maneuver in women affected by endometriosis.

	LHA			AP			LRT		
	Rest	Contraction	Valsalva	Rest	Contraction	Valsalva	Rest	Contraction	Valsalva
Raimondo et al. (2017)^a^	Area (cm^2^) 12.0 ± 2.8 vs. 13.2 ± 2.2 *p* = 0.03	Area (cm^2^) 17.4 ± 10.1 vs. 22.4 ± 9.3 *p* < 0.001 Reduction (%) 17.4 ± 10.1 vs. 22.4 ± 9.3 *p* < 0.001	Area (cm^2^) 14.5 ± 4.3 vs. 17.3 ± 4.6 *p* < 0.01 Increase (%) 12.6 ± 16.0 vs. 43.2 ± 31.9 *p* = 0.01	NS	Diameter (cm) NS Reduction (%) 9.2 ± 4.6 vs. 18.9 ± 10 *p* = 0.001	Diameter (cm) 5.0 ± 0.8 vs. 5.5 ± 0.8 *p* < 0.01 Increase (%) NS	NS	Diameter (cm) NS Reduction (%) 3.7 ± 2.7 vs. 10.7 ± 8.6 *p* = 0.01	NS
Mabrouk et al. (2018)^a^	Area (cm^2^) 10.8 ± 2.6 vs. 11.9 ± 2.5 *p* = 0.03	Area (cm^2^) 8.8 ± 1.9 vs. 9.3 ± 2.1 *p* = 0.03 Reduction (%) 16.0 ± 13.9 vs. 21.9 ± 10.4 *p* = 0.04	Area (cm^2^) 12.2 ± 2.8 vs. 14.2 ± 3.7 *p* = 0.02 Increase (%) NS	NS	NS	NS	Diameter (cm) 3.3 ± 0.4 vs. 3.6 ± 0.6 *p* = 0.003	Diameter (cm) 3.0 ± 0.4 vs. 3.7 ± 0.5 *p* = 0.009 Reduction (%) NS	Diameter (cm) 3.4 ± 0.4 vs. 3.8 ± 0.7 *p* = 0.02 Increase (%) NS
Raimondo et al. (2022)^b^	NS	NA	Area (cm^2^) 12.6 ± 3.2 vs. 14.6 ± 4.4 *p* = 0.041 Increase (%) NS	NS	NA	Diameter (cm) 5.0 ± 0.7 vs. 5.3 ± 0.9 *p* = 0.047 Increase (%) NS	NA	NA	NA
Mabrouk et al. (2019)^c^	NS	NS	NS	NS	NS	LAM coactivation, *n* (%) 31 (64.6%) vs. 19 (31.7%) *p* < 0.001	NS	NS	NS
Mabrouk et al. (2020)^d^	Area (cm^2^) 11.0 ± 2.4 vs. 12.0 ± 2.4 *p* = 0.014	NA	NA	NA	NA	NA	NA	NA	NA
Arena et al. (2022)^a^	NA	NA	NA	NA	Diameter narrowing (%) in DIE 14.6 ± 10.4% (0.0–37.1) *p* < 0.001	NA	NA	NA	NA
Del Forno et al. (2020)^e^	Area (cm^2^) 11.5 ± 2.4 vs. 10.4 ± 2.1 *p* = 0.0395	Area (cm^2^) 10.5 ± 2.1 vs. 9.5 ± 3.0 *p* = 0.0278	Area (cm^2^) 14.0 ± 2.5 vs. 12.0 ± 2.2 *p* = 0.0293	NA	NA	NA	NA	NA	NA
Del Forno et al. (2021)^f^	NS	NS	Change (%) 20.0 ± 24.8% vs. −0.5% ± 3.3% *p* = 0.02	NA	NA	NA	NA	NA	NA

Abbreviations: AP, anteroposterior diameter; LAM, levator ani muscle; LHA, levator hiatal area; LRT, left–right transverse diameter; NA, not available; NS, not statistically significant.

^a^ Women with endometriosis versus healthy controls.

^b^ Women with endometriosis and constipation versus no constipation.

^c^ Women with posterior DIE and voiding dysfunction versus no voiding dysfunction.

^d^ Women with endometriosis and superficial dyspareunia versus no superficial dyspareunia.

^e^ Women with endometriosis and dyspareunia before and after pelvic floor physiotherapy.

^f^ Women with endometriosis and superficial dyspareunia after pelvic floor physiotherapy versus controls.

Raimondo et al. (2017) published a pilot prospective study in 2017 evaluating the static and dynamic morphometry of PFM using 3D‐4D TPU in women with deep infiltrating endometriosis (DIE, *N* = 50 nulliparous women) compared with asymptomatic healthy women (*n* = 35 nulliparous women). LHA and anteroposterior (APD) and left–right transverse (LRTD) diameters at rest, on maximum PFM contraction and under maximum Valsalva maneuver were evaluated. Women with DIE had a smaller LHA at rest (*p* = 0.03) and during Valsalva maneuver (*p* < 0.01). Furthermore, reduction of LHA during PFM contraction (*p* < 0.001) and enlargement of LHA from baseline during Valsalva maneuver (*p* = 0.01) were significantly less marked.

These findings were confirmed in another prospective study by Mabrouk et al. (2018). Morphometry of PFM in women affected by ovarian endometriosis with DIE was analyzed. Aim of the study was to assess the impact of DIE retroperitoneal infiltration on PFM function. 114 patients with ovarian endometriosis, 75 with DIE and 39 without DIE were enrolled. Compared with controls, women with DIE showed a smaller LHA at rest (*p* = 0.03), during maximum voluntary contraction (*p* = 0.03) and under Valsalva maneuver (*p* = 0.02) and a smaller reduction in LHA during PFM contraction (*p* = 0.04).

Two studies analyze the correlation between PFM dysfunction and gastrointestinal symptoms such as voiding alteration and chronic constipation (CC) in women with posterior deep endometriosis.

Raimondo et al. (2022) published an observational prospective cohort study with the aim to assess the association between sonographic findings at TPU and CC in women with endometriosis. 87 women were enrolled: 29 (33%) with CC and 58 (67%) without CC. Women underwent TPU at rest and during Valsalva maneuver; LHA, APD, and levator ani muscle (LAM) coactivation were evaluated. Ultrasound findings were compared between women with and without CC in the whole study population and subsequently in two subgroups (only ovarian endometriosis and deep infiltrating endometriosis). Women with endometriosis and CC showed a smaller LHA during Valsalva (*p* = 0.041), reduction of LHA (*p* = 0.025), and APD (*p* = 0.035) enlargement from rest to maximum Valsalva. A higher prevalence of LAM coactivation compared with women without CC was observed (65.5% versus 18.9%; *p* < 0.001). The 87 patients enrolled in this study were divided into two subgroups: isolated ovarian endometriosis (*n* = 42, 48%) and DIE (*n* = 45, 52%). In the DIE subgroup, no significant difference was demonstrated between women with and without CC. In the ovarian endometriosis subgroup, women with CC had smaller LHA (*p* = 0.037) and APD (*p* = 0.047) under Valsalva maneuver, less enlargement of LHA (*p* = 0.002) and APD (*p* < 0.001) from rest to maximum Valsalva and a higher prevalence of LAM coactivation (75% versus 6.7%; *p* < 0.001) compared to non‐CC patients.

Mabrouk et al. (2019) conducted a prospective study on 108 symptomatic (voiding dysfunction—VD) women scheduled for surgical removal of posterior DIE in order to evaluate the correlation between the presence of VD and altered PFM morphometry assessed due to 3D/4D TPU. Women with VD showed a higher rate of LAM coactivation compared with women without VD [64.6% (31/48) versus 31.7% (19/60), respectively; *p* < 0.001].

Another symptom strongly associated with deep endometriosis is dyspareunia, defined as painful sexual intercourse. Women with endometriosis may experience sexual impairments, with negative consequences on their psychological and physical health and quality of life, especially considering the chronic nature of the disease and its association with infertility.

Four studies analyze the role of TPU in women affected by dyspareunia and DIE.

Mabrouk et al. (2020) conducted an observational study enrolling 160 symptomatic, sexually active women with clinical and sonographic diagnosis of DIE or isolated ovarian endometriosis.

LHA was significantly narrower in women with superficial dyspareunia than in women without superficial dyspareunia (*p* = 0.014).

Arena et al. (2022) conducted a prospective study on nulliparous women affected by endometriosis. Patients were divided into two subgroups: ovarian endometriosis (OE) and DIE. 246 patients were considered for the final study (*n* = 82 healthy controls, *n* = 82 OE, *n* = 82 DIE).

All patients underwent 3D/4D TPU. APD measurements, both at rest and during maximal PFM contraction, were obtained. The difference of APD from rest to maximal contraction was calculated as percent change from baseline (ΔAPD), and patients were categorized using the 4‐point pelvic contraction scale [1: absent (< 7%), 2: weak (7%–17.9%), 3: normal (18%–34.9%), 4: strong (≥ 35%)].

Mean difference in APD between relaxed state and maximal PFM contraction was 23.3% ± 7.9% (range 2.4–40.0) in healthy controls, 20.5% ± 9.0% (range 0.0–37.3) in patients with OE, and 14.6% ± 10.4% (range 0.0–37.1) in patients with DIE (*p* < 0.001). Moreover, a significant negative correlation between the contraction scale and dyspareunia (*p* = 0.032) was discovered, and this result appeared to be stronger among patients with DIE (*p* = 0.076). Dyspareunia is associated with low PFM contractility. Indeed, women with DIE have an increased PFM tone at rest; on the other hand, they have a decreased strength during voluntary contraction.

Two studies evaluate the utility of TPU to identify the effects of pelvic floor physiotherapy (PFP) in patients with endometriosis and dyspareunia.

Del Forno et al. (2020) conducted a prospective study to evaluate the effects of pelvic floor physiotherapy in women with DIE and associated dyspareunia. 10 nulliparous women aged between 18 and 45 years with a clinical and sonographic diagnosis of DIE and associated dyspareunia were enrolled. Women underwent Thiele massage, a technique involving targeted digital pressure followed by controlled stretching of the pelvic musculature. LHA measurements before and after physiotherapy were compared. The mean post‐therapy LHA was significantly larger at rest (*p* = 0.0395), under maximum PFM contraction (*p* = 0.0278), and under maximum Valsalva maneuver (*p* = 0.0293).

Subsequently, Del Forno et al. (2021) conducted a randomized controlled trial on 34 nulliparous women diagnosed with DIE and associated superficial dyspareunia.

All patients underwent 3D/4D TPU and LHA at rest, under maximum PFM contraction and under maximum Valsalva maneuver. Patients were asked to rate their pain symptoms using a numerical rating scale (NRS). Patients were assigned randomly (1:1 ratio) to no intervention (control group, 17 women) or treatment with five individual sessions of PFP (study group, 17 women). All women underwent a second evaluation of pain symptoms and LHA on TPU 4 months after the first examination. The percentage change in LHA on maximum Valsalva maneuver between the two examinations was higher in the study group than in the control group (*p* = 0.02), indicating better PFM relaxation. Moreover, after PFP treatment, the NRS score for superficial dyspareunia remained almost unchanged in the control group (Δ‐NRS 0), while a marked reduction in the study group was observed (*p* < 0.01).

### 3.4 Vulvodynia

Six studies (Morin et al. 2014; McLean et al. 2016; Thibault‐Gagnon et al. 2016; Fontaine et al. 2018; Bardin et al. 2019, 2020) reported the use of TPU in patients with vulvodynia. Main results are summarized in Table 6.

**TABLE 6:** Pelvic floor muscle (PFM) morphometry on three‐ and four‐dimensional transperineal ultrasound (3D‐4D TPU) at rest, on PFM contraction, and on Valsalva maneuver in women affected by vulvodynia.

	LHA			LPA			LRT			ARA		
	Rest	Contraction	Valsalva	Rest	Contraction	Valsalva	Rest	Contraction	Valsalva	Rest	Contraction	Valsalva
Morin et al. (2014)^a^	Area (cm^2^) 9.77 ± 1.88 vs. 10.83 ± 2.22 *p* = 0.011	Area narrowing (%) 16.06 ± 9.58 vs. 27.12 ± 12.14 *p* < 0.001	NA	Angle (°) 29.9 ± 6.35 vs. 26.7 ± 6.4 *p* = 0.013	Angle (°) 37.05 ± 10.39 vs. 44.12 ± 10.69 *p* = 0.001	NA	NS	Diameter (cm) 3.22 ± 0.42 vs. 3.07 ± 0.33 *p* = 0.044	NA	Angle (°) 104.97 ± 13.41 vs. 117.35 ± 8.77 *p* < 0.001	Angle excursion (°) 7.94 ± 8.56 vs. 15.34 ± 8.58 *p* < 0.001	NA
McLean et al. (2016)^a^	NA	NA	NA	Smaller in PVD than controls (*p* = 0.001)	Smaller in PVD than controls (*p* = 0.001)	Smaller in PVD than controls (*p* = 0.001)	NA	NA	NA	NS	Smaller than at rest *p* = 0.022	Smaller than at rest *p* = 0.022
Thibault‐Gagnon et al. (2016)^a^	Area (cm^2^) 11.02 ± 2.38 vs. 12.38 ± 2.64 *p* = 0.020	Area (cm^2^) 7.95 ± 1.80 vs. 8.92 ± 1.75 *p* = 0.021	Area (cm^2^) 15.49 ± 4.06 vs. 18.91 ± 5.18 *p* = 0.004	NA	NA	NA	NS	NS	Diameter (cm) 3.97 ± 0.46 vs. 4.37 ± 0.47 *p* = 0.001	NA	NA	NA
Fontaine et al. (2018)^b^	NS	NS	NA	NS	NS	NA	NS	NS	NA	NS	NS	NA
Bardin et al. (2019)^c^	NS	Larger in TCA + KTP group than at baseline *p* < 0.05	NS	NS	NS	NS	NS	NS	Larger in TCA + KTP group than at baseline *p* < 0.05	More obtuse in TCA + KTP group than in TCA group *p* = 0.022	More obtuse in TCA + KTP group than in TCA group *p* = 0.007	More obtuse in TCA + KTP group than in TCA group *p* = 0.013
Bardin et al. (2020)^c^	NA	Greater narrowing in TCA + KTP group than at baseline *p* = 0.04	NA	NA	Angle excursion (°) 13.97 ± 10 vs. 8.75 ± 13.7 *p* = 0.02	NA	NA	NA	NA	NS	NA	NA

Abbreviations: ARA, anorectal angle; KTP, kinesiotherapy protocol; LHA, levator hiatal area; LPA, levator plate angle; LRT, left–right transverse diameter; NA, not available; NS, not statistically significant; PVD, provoked vestibulodynia; TCA, tricyclic antidepressant.

^a^ Women with provoked vulvodynia versus healthy controls.

^b^ Women with primary versus secondary provoked vestibulodynia.

^c^ Women with vulvodynia submitted to tricyclic antidepressant versus women with vulvodynia submitted both tricyclic antidepressant and kinesiotherapy.

Morin et al. (2014) enrolled 49 patients affected by provoked vestibulodynia (PVD) and 51 healthy controls. 4D TPU at rest and under maximal pelvic contraction was performed. Compared to controls, patients with PVD at rest had a larger levator plate angle (*p* = 0.013), a more acute anorectal angle (*p* < 0.001), a smaller LHA (*p* ≤ 0.028), and a smaller AP diameter (*p* ≤ 0.028). Under PFM contraction, women affected by PVD showed less displacement of the bladder neck (*p* < 0.001), lower excursion of the levator plate (*p* < 0.001), and anorectal angle (*p* < 0.001) compared to healthy controls. LHA was significantly modified in PVD under contraction. In fact, data from the study showed less LHA narrowing (*p* < 0.001), less levator hiatus AP (*p* < 0.001), and LRT (*p* < 0.001) diameter reduction.

McLean et al. (2016) and Thibault‐Gagnon et al. (2016) added the evaluation of PFM under Valsalva maneuver. 4D TPU at rest, under maximal pelvic contraction and Valsalva maneuver was performed. The examiners acquired 3 consecutive pelvic volumes at each stage. The authors analyzed the same population. In this review only the different data emerged from the two studies are reported. 38 women with PVD and 39 healthy control women were enrolled. Data from six patients with PVD and six controls were not analyzed because they could not perform a maximum contraction of the pelvic floor or an adequate Valsalva maneuver.

McLean et al. (2016) showed that women with PVD had smaller levator plate length (LPL) than controls in all states (rest, contraction, and Valsalva) and it became shorter under maximal voluntary contraction and longer under Valsalva maneuver (*p* = 0.014). Anorectal angle (ARA) in PVD became smaller than at rest during contraction and Valsalva maneuver (*p* = 0.022) and levator plate angle (LPA) is smaller than controls in all states and became larger under maximal voluntary contraction and smaller under Valsalva maneuver (*p* = 0.001). Finally, the authors found that bladder neck displacement during Valsalva maneuver was significantly lower in PVD than controls (*p* = 0.001).

Thibault‐Gagnon et al. (2016) showed a significantly smaller LHA at rest (*p* = 0.020), under maximum contraction (*p* = 0.021), and under Valsalva maneuver (*p* = 0.004) in women with PVD compared to healthy controls. During the Valsalva maneuver, LRT diameter showed a significant reduction in women affected by PVD (*p* = 0.001).

In 2018, Fontaine et al. (2018) evaluated whether there could be different ultrasound characteristics of PFM in patients with primary (PVD1) and secondary (PVD2) provoked vulvodynia. A total of 212 nulliparous premenopausal women were included in this study. Morphometry and functionality of PFM were respectively analyzed through 3D/4D TPU (at rest and under maximum contraction) and a pediatric‐size dynamometric speculum. Ultrasound parameters taken into account were the following: LHA, AP, and LRT diameters. No significant differences between the two groups were found. These results show that PFM alterations in PVD are not impacted by when the symptoms appear and could both benefit from the same treatment.

Internationally, there is no standardized treatment for vulvodynia and a multimodal therapeutic approach has been proposed from several guidelines. Treatment involves a step‐by‐step approach with the aim of treating the possible triggers of vulvar symptoms. (Falsetta et al. 2017; Goldstein et al. 2016).

Among treatments proposed in literature, physical therapy has emerged especially in PVD with the aim to rehabilitate PMF and it could be used in conjunction with other treatments such as pharmacological therapy (antidepressants and anticonvulsants) (Bergeron et al. 2020; Morin, Carroll, and Bergeron 2017). The most commonly used physical treatment methods are electromyography (EMG) biofeedback, electrotherapy, manual therapy with stretching and massage of the pelvic floor, kinesiotherapy (KTP), which involves the self‐administered performance of voluntary muscle contraction and relaxation exercises in various body positions, health education, and the use of dilators (Hartmann et al. 2007).

Bardin et al. (2019, 2020) investigated PFM biometric changes in women with PVD after physical therapy by using 3D/4D TPU.

In 2019, Bardin et al. (2019) conducted a single‐blinded randomized controlled trial study on 86 women with vulvodynia submitted to pharmacological treatment with tricyclic antidepressant (TCA) or both TCA and kinesiotherapy. Outcomes were evaluated at rest, under Valsalva maneuver, and maximal contraction at baseline and after 8 weeks of therapy.

After 8 weeks, TCA and kinesiotherapy group had significant increase of AP diameter at rest (*p* = 0.008) and under contraction (*p* = 0.004), more obtuse levator plate angle at rest (*p* = 0.022) and under contraction (*p* = 0.007), larger LHA under contraction (*p* < 0.05), larger LRT diameter (*p* < 0.05) and more obtuse anorectal angle (*p* = 0.013) under Valsalva maneuver. Control group treated with only TCA did not have significant changes from baseline (*p* > 0.05). These results show how kinesiotherapy can be a useful tool in PVD patients by determining significant changes in PFM activity.

These data were subsequently confirmed in a second single‐blinded randomized controlled trial conducted in 2020 by Bardin et al. (2020) on a sample of 57 women affected by vulvodynia. 30 patients were randomized to kinesiotherapy plus TCA (amitriptyline 25 mg) and 27 patients to the control group (amitriptyline 25 mg only). All patients underwent 4D TPU at baseline and after 8 weeks of treatment and the examination was conducted at rest and under voluntary maximum contraction.

From baseline to 8 weeks, the KTP plus TCA group showed a greater symphysis levator distance at rest (*p* = 0.008) and a higher LHA narrowing (*p* = 0.04). Also, in this study, the control group showed no statistically significant changes in PFM biometrics pre and post‐treatment.

### 3.5 Gynecological Cancer

Three studies (De Morais Siqueira et al. 2021; Cyr et al. 2021, 2022) reported the use of TPU in patients with gynecological cancer. Main results are summarized in Tables 7 and 8.

**TABLE 7:** Pelvic floor muscle (PFM) morphometry on three‐ and four‐dimensional transperineal ultrasound (3D‐4D TPU), at rest and on maximum voluntary PFM contraction, within gynecological cancer patients after pelvic radiotherapy (RT).

	Change in symphysis‐levator distance				Change in puborectalis muscle thickness			
	CC vs. EC	Interval after RT (months)	Cancer staging	Menopause duration	CC vs. EC	Interval after RT (months)	Cancer staging	Menopause duration^a^
De Morais Siqueira et al. (2021)	NS	NS	I 0.80 ± 0.44 II 0.55 ± 0.047 III 0.36 ± 0.58 *p* = 0.028	NS	Left lower PR 0.20 ± 0.18 vs. 0.09 ± 0.19 *p* = 0.039 right lower PR 0.19 ± 0.18 vs. 0.07 ± 0.17 *p* = 0.014	Left upper PR (< 60) 0.17 ± 0.15 vs. (≥ 60) 0.09 ± 0.14 *p* = 0.029 Right upper PR (< 60) 0.20 ± 0.17 vs. (≥ 60) 0.08 ± 0.17 *p* = 0.013	NS	Left upper PR *p* = 0.0075 Right upper PR *p* = 0.0029

*Note:* “Change” refers to the difference between rest and muscle contraction.

Abbreviations: CC, cervical cancer; EC, endometrial cancer; NS, not statistically significant; PR, puborectalis muscle.

^a^ Women with shorter menopause time versus longer menopause time.

**TABLE 8:** Pelvic floor muscle (PFM) morphometry on three‐ and four‐dimensional transperineal ultrasound (3D‐4D TPU), at rest and on maximum voluntary PFM contraction, in gynecological cancer survivors with dyspareunia.

	LHA			LPA			ARA		
	Rest	Contraction	Calculated excursion	Rest	Contraction	Calculated excursion	Rest	Contraction	Calculated excursion
Cyr et al. (2021)^b^	Area (cm^2^) 13.87 ± 3.79 vs. 17.74 ± 3.44 *p* < 0.001	Area (cm^2^) 10.87 ± 2.78 vs. 12.47 ± 2.34 *p* = 0.01	Area narrowing (%) 20.13 ± 8.87 vs. 28.78 ± 10.52 *p* = 0.02	NS	NS	NS	Angle (°) 106.78 ± 9.40 vs. 111.99 ± 7.44 *p* = 0.01	NS	Angle (°) 5.85 ± 5.50 vs. 10.45 ± 6.65 *p* = 0.03
Cyr et al. (2022)^a^	Area (cm^2^) 13.4 ± 3.0 vs. 15.4 ± 3.4 Change 2.0 ± 2.5 *p* < 0.001	Area (cm^2^) 10.7 ± 2.1 vs. 10.2 ± 2.2 Change −0.6 ± 1.3 *p* = 0.032	Area narrowing (%) 18.9 ± 9.2 vs. 32.9 ± 10.1 Change 14.4 ± 9.8 *p* < 0.001	Angle (°) 23.6 ± 6.6 vs. 20.0 ± 6.6 Change −3.9 ± 4.7 *p* < 0.001	NS	Angle (°) 8.6 ± 5.9 vs. 13.5 ± 8.3 Change 5.5 ± 7.4 *p* < 0.001	Angle (°) 106.8 ± 9.8 vs. 112.6 ± 8.9 Change 7.5 ± 6.3 *p* < 0.001	NS	Angle (°) 4.9 ± 5.8 vs. 14.0 ± 8.3 Change 10.2 ± 10.0 *p* < 0.001

*Note:* “Change” refers to the difference between baseline and post‐treatment morphometry.

Abbreviations: ARA, anorectal angle; LHA, levator hiatal area; LPA, levator plate angle; NS, not statistically significant.

^a^ Gynecological cancer survivors with dyspareunia before and after pelvic floor multimodal physiotherapy.

^b^ Gynecological cancer survivors with dyspareunia versus asymptomatic women.

De Morais Siqueira et al. (2021) conducted a cross‐sectional study that included 64 women (26 with cervical cancer and 38 with endometrial cancer treated with radiotherapy (RT) for at least 6 months with or without previous surgery), with the aim to assess PFM function through digital palpation and 4D TPU.

The ultrasonographic variables analyzed were: levator plate angle, hiatal area, puborectalis muscle thickness, puborectalis strain and levator ani muscle integrity. Women who finished RT with < 60 months displayed higher thickening of the left (*p* = 0.029) and right (*p* = 0.013) upper portion of the puborectalis muscle during muscle contraction. They also observed that, regarding cancer staging, women with Stage I cancer presented better PFM function related to higher narrowing of the distance between the pubic symphysis and levator ani (*p* = 0.028). Moreover, longer menopause duration was correlated with smaller narrowing of the distance between the pubic symphysis to levator ani (*p* = 0.002), hiatal area (*p* = 0.039) and thickening of the left (*p* = 0.0003) and right (*p* = 0.002) upper puborectalis muscle during PFM contraction, demonstrating worse PFM function. For instance, a shorter RT interval, shorter menopause duration and early cancer stage were associated with ultrasound parameters that indicate greater muscle thickness and an indirectly better PFM function. The limit of this study is that the variables analyzed have not been divided according to the neoplastic site.

Moreover, in addition to pelvic floor disorders, gynecological cancer survivors are more prone to experience sexual dysfunction. Cohort studies showed that 55%–67% of gynecological cancer survivors have pain during sexual intercourse, also known as dyspareunia (Rutledge et al. 2010; Jensen et al. 2003; Stinesen Kollberg et al. 2015).

Cyr et al. (2021) conducted a comparative cross‐sectional study on 24 gynecological cancer survivors with dyspareunia and 32 asymptomatic women with a history of total hysterectomy. Patients underwent a 3D/4D TPU in order to assess the PFM morphometry (bladder neck position, levator plate angle, anorectal angle, and levator hiatal dimensions). Gynecological cancer survivors with dyspareunia presented smaller hiatal area (*p* < 0.001) and smaller anorectal angle (*p* = 0.01) at rest, implying a heightened PFM tone. Moreover, these patients presented fewer changes from rest to maximal contraction for anorectal angle (*p* = 0.01) and levator hiatal dimensions (*p* = 0.002), suggesting an elevated tone or altered contractile properties.

For instance, the same research group directed by Cyr MP. conducted a prospective interventional study (Cyr et al. 2022) on 31 gynecological cancer survivors with dyspareunia with the aim to investigate the changes in pelvic floor morphometry and muscle function after multimodal pelvic floor physiotherapy treatment. Women were assessed at baseline and post‐treatment. Pelvic floor morphometry was evaluated at rest and under maximal voluntary contraction and bladder neck position, LHA, anorectal and levator plate angles were evaluated with 3D/4D TPU. After physiotherapy treatment at rest, the anorectal angle (*p* < 0.001) and the LHA increased (*p* < 0.001) while the levator plate angle decreased (*p* < 0.001), suggesting a reduction in muscle tone. In addition, a reduction in LHA (*p* = 0.032) and levator hiatal anterior–posterior diameter (*p* = 0.022) was found from baseline to post‐treatment on maximal contraction, suggestive of improved pelvic floor muscle contractility. Moreover, the parameters assessing the changes from rest to maximal contraction significantly improved (*p* < 0.001), supporting the hypothesis of decreased muscle tone and improved muscle contractility following treatment. For instance, due to these results, it is possible to consider multimodal physiotherapy the key treatment in order to reduce dyspareunia in this population.

## 4 Discussion

This study explores the primary applications of TPU, focusing on its role in the evaluation of deep infiltrating endometriosis, vulvodynia, and gynecological tumors.

Endometriosis is a chronic inflammatory disease and is defined as the presence of endometrial glands and stroma outside the uterine cavity. It affects 6%–10% of women of reproductive age (Clement 2007; Giudice 2010). DIE occurs when endometriotic implants penetrate below the peritoneal surface, involving the rectovaginal septum, rectum, uterosacral ligaments, ureters, and urinary bladder (Chapron et al. 2006; Seracchioli et al. 2008) DIE is strongly associated with severe pelvic pain, including dysmenorrhea, dyspareunia, CPP, dysuria, and dyschezia, as well as bladder, sexual, and bowel dysfunctions (Cornillie et al. 1990) Moreover, CPP syndromes, such as endometriosis, can be associated with increased pelvic floor muscle tone, suggesting an important role for the pelvic floor in the pathogenesis of pain (Butrick 2009; Aredo et al. 2017). The studies reviewed highlight the utility of three‐dimensional/four‐dimensional transperineal ultrasound (3D/4D TPU) in evaluating PFM morphometry and function in women with DIE, providing critical insights into the pathophysiology of pain and related symptoms. The findings consistently show that women with DIE exhibit reduced LHA measurements at rest, during PFM contraction, and under Valsalva maneuver, as well as diminished muscle relaxation and strength. These alterations contribute to increased pelvic floor muscle tone and coactivation, exacerbating pain and functional impairments. Furthermore, PFM dysfunction was correlated with gastrointestinal and sexual symptoms, highlighting the role of PFM in the complex symptomatology of DIE. TPU was also shown to be a valuable tool in monitoring therapeutic interventions, such as pelvic floor physiotherapy, which significantly improved PFM relaxation and reduced pain symptoms, particularly dyspareunia. These findings emphasize the importance of incorporating PFM assessment and targeted therapeutic approaches in the clinical management of DIE to improve quality of life and pain outcomes in affected women.

Vulvodynia is described as persistent vulvar pain lasting at least 3 months without an identifiable cause, which may have potential associated factors. These include musculoskeletal (e.g., pelvic muscle overactivity) and neurological (central and peripheral) factors, comorbid pain syndromes (such as fibromyalgia, irritable bowel syndrome, painful bladder syndrome), structural defects (e.g., perineal descent), psychosocial factors, inflammation, genetic and hormonal factors (Bornstein et al. 2016).

Vulvodynia is classified into several categories that can be divided by location (localized such as vestibulodynia or clitorodynia, generalized or mixed), onset (primary, secondary), temporal pattern (intermittent, persistent, constant, immediate, delayed), and trigger (provoked, spontaneous, or mixed). The most common subtype of vulvodynia is localized provoked vestibulodynia and is associated with an algic trigger such as touch, penetration, or clothes.

The etiopathogenesis of vulvodynia is determined by both biomedical (e.g., peripheral and central pain mechanisms, hormonal factors, pelvic floor muscle dysfunction, neuroinflammatory factors) and psychosocial (e.g., anxiety and depression, sexual motivation) factors (Bergeron et al. 2020).

Controlled studies have demonstrated abnormalities of the PFM at rest (hypertonicity, poor muscle control, hypersensitivity) and under volunteer contraction. Several validated diagnostic techniques are being used recently to assess PFM dysfunction in patients affected by vulvodynia, including 3D/4D TPU (Morin, Binik, et al. 2017). The use of TPU in patients with vulvodynia demonstrates significant alterations in pelvic floor muscle morphometry and function compared to healthy controls. Studies consistently show that patients with provoked vulvodynia exhibit altered levator hiatus area, levator plate angles, and bladder neck displacement at rest, under contraction, and during Valsalva maneuvers. Importantly, kinesiotherapy combined with pharmacological treatment significantly improves PFM parameters, supporting its role as an effective intervention in vulvodynia management. These findings underline the value of TPU in assessing PFM dysfunction and monitoring therapeutic outcomes in vulvodynia patients.

4D TPU is increasingly utilized in urogynecology to assess pelvic floor muscle dysfunctions, including in women with gynecological cancer, particularly those with cervical or endometrial cancer who have undergone pelvic radiotherapy. RT, including external beam radiation therapy (EBRT) and brachytherapy, can negatively affect PFM function, leading to decreased muscle contractility, altered sensitivity, pain, genitourinary symptoms, vaginal stenosis, and psychological changes (Noronha et al. 2013; Bernard et al. 2016, 2017). TPU in gynecological cancer patients provides valuable insights into PFM dysfunction, highlighting the impact of cancer treatments, with findings suggesting altered PFM morphometry, increased muscle tone, and reduced contractility, contributing to conditions like dyspareunia. Multimodal pelvic floor physiotherapy has shown effectiveness in reducing muscle tone and enhancing contractility, alleviating dyspareunia symptoms, thus emphasizing the need for physiotherapy in managing pelvic floor disorders in this population.

## 5 Conclusion

TPU is emerging as an invaluable diagnostic tool for assessing anatomical and functional alterations associated with pelvic floor disorders. The studies reviewed herein demonstrate the broad applicability of TPU in various conditions contributing to CPP, including endometriosis and vulvodynia, and during the treatment of gynecological cancers. However, the current use of TPU has been limited to evaluating only certain etiologies of CPP, highlighting a need for expanded research into its utility across other clinical conditions. Additionally, the small sample sizes in many of these studies necessitate further investigations with larger cohorts to validate the findings.

This review underscores the strengths of TPU, including its reproducibility, dynamic imaging capabilities, absence of ionizing radiation, and cost‐effectiveness. These attributes position TPU as a diagnostic modality that is likely to become increasingly integrated into clinical practice for the evaluation of pelvic floor muscle alterations in patients with CPP. Future research should focus on expanding its applications and confirming its diagnostic value across diverse patient populations.

## Funding

The authors have nothing to report.

## Conflicts of Interest

The authors declare no conflicts of interest.

## Supporting information


**Supplementary Appendix A.** Example of search strategy (PubMed).

## Data Availability

The data supporting the findings of this study are fully included within the article.
