# Supplementary material for: Transperineal Ultrasound in Chronic Pelvic Pain: A Systematic Review
Source: J Clin Ultrasound. 2026 Sep 14;54(9):2252–68. doi: 10.1002/jcu.70406 (PMC13730181; doi:10.1002/jcu.70406)
Supplement: Supplementary file 1 [file JCU-54-2252-s001.docx]

**Supplementary Appendix A. Example of Search Strategy (PubMed)**

The systematic review was conducted in accordance with the Preferred Reporting Items for Systematic Reviews and Meta-Analyses (PRISMA) guidelines. A comprehensive literature search was electronically performed and updated through July 2025 across the following databases:

- **PubMed**
- **Medline**
- **ScienceDirect**
- **EMBASE**
- **Cochrane Central Register of Controlled Trials (CENTRAL)**

The search strategy involved the use of Medical Subject Headings (MeSH), keywords, and word variants related to the following core concepts:

- **Transperineal ultrasound**
- **Chronic pelvic pain**

These terms were combined using Boolean operators (AND, OR) to maximize sensitivity. The search strategy used for PubMed is detailed below and was adapted accordingly for the other databases.

Search String (PubMed): ("transperineal ultrasound" OR "perineal ultrasound" OR "TPU") AND ("chronic pelvic pain" OR "pelvic pain" OR "CPP").

No restrictions were applied regarding the publication date due to the recent introduction of TPU in routine gynecological assessment. The following filters were applied:

- **Language:** English
- **Study type:** Clinical trials, observational studies, prospective and retrospective studies

In addition:

- Conference abstracts were screened for relevant content.
- Reference lists of all included articles were manually checked to identify additional studies.
- Titles and abstracts were screened for relevance.
- Full texts of potentially eligible studies were retrieved and assessed for final inclusion.
